# Evaluation and management of chemotherapy-induced cardiotoxicity in breast cancer: a Delphi study

**DOI:** 10.1007/s12094-016-1508-y

**Published:** 2016-04-21

**Authors:** J. Gavila, M. Á. Seguí, L. Calvo, T. López, J. J. Alonso, M. Farto, R. Sánchez-de la Rosa

**Affiliations:** 1 Servicio de Oncología Médica, Fundación Instituto Valenciano de Oncología, Calle del Profesor Beltrán Bàguena, 8, 46009 Valencia, Spain; 2 Servicio de Oncología Médica, Corporació Sanitaria ParcTaulí, Barcelona, Spain; 3 Servicio de Oncología Médica, Complejo Hospitalario Universitario A Coruña, A Coruña, Spain; 4 Servicio de Cardiología, Hospital Universitario La Paz, Madrid, Spain; 5 Servicio de Cardiología, Hospital Universitario de Getafe, Madrid, Spain; 6 Medical Department, TEVA Pharma, Madrid, Spain

**Keywords:** Delphi study, Cardiotoxicity, Breast cancer, Anthracycline, Trastuzumab

## Abstract

**Purpose:**

While much progress has been made in the treatment of breast cancer, cardiac complications resulting from therapy remain a significant concern. Both anthracyclines and novel targeted agents can inflict cardiac damage. The present study aimed to evaluate the difference between what it is currently done and what standards of care should be used to minimizing and managing cardiac toxicity in breast cancer survivors.

**Methods:**

A two-round multicenter Delphi study was carried out. The panel consisted of 100 oncologists who were asked to define the elected therapies for breast cancer patients, the clinical definition and patterns of cancer drug-derived cardiac toxicity, and those protocols focused on early detection and monitoring of cardiovascular outcomes.

**Results:**

Experts agreed a more recent definition of cardiotoxicity. Around 38 % of patients with early-stage disease, and 51.3 % cases with advanced metastatic breast cancer had preexisting risk factors for cardiotoxicity. Among risk factors, cumulative dose of anthracycline ≥450 mg/m^2^ and its combination with other anticancer drugs, and a preexisting cardiovascular disease were considered the best predictors of cardiotoxicity. Echocardiography and radionuclide ventriculography have been the proposed methods for monitoring changes in cardiac structure and function. Breast cancer is generally treated with anthracyclines (80 %), so that the panel strongly stated about the need to plan a strategy to managing cardiotoxicity. A decline of left ventricular ejection fraction (LVEF) >10 %, to an LVEF value <53 % was suggested as a criterion for changing the dose schedule of anthracyclines, or suspending the treatment of chemotherapy plus trastuzumab until the normalization of the left ventricular function. The use of liposomal anthracyclines was strongly suggested as a treatment option for breast cancer patients.

**Conclusions:**

The present report is the first to produce a set of statements on the prevention, evaluation and monitoring of chemotherapy-induced cardiac toxicity in breast cancer patients.

## Introduction

Breast cancer is the commonest cancer in females, being responsible for 16 % of all cancer cases worldwide. A report of the World Health Organization showed that breast cancer causes 1.6 % of all annual deaths in the world [1]. Since the 1970s, major advances in screening technologies and treatment options have resulted in significant declines in breast cancer mortality [2]. Treatment options can be classified into two groups: conventional therapies (i.e., chemotherapy and radiotherapy) which aim to have a greater effect on cancer cells without being specifically directed to them; and newer molecular targeted therapies (i.e., trastuzumab), which are designed to recognize determined cancer cell markers in order to spare normal cells [3]. However, their clinical utility is limited by cumulative, dose-related progressive myocardial damage [4, 5]. Taxanes, alkylating agents (i.e., cisplatin), antimetabolites (i.e., capecitabine), mitoxantrone, trastuzumab, bevacizumab, and the tyrosine kinase inhibitor, sunitinib have shown cardiac toxic effects [6, 7].

Cardiac toxicity associated with cancer treatment is a growing source of significant morbidity and mortality, and may vary from subclinical myocardial dysfunction to irreversible heart failure (HF) or even death [8]. Cumulative doses and concomitant use of adjuvant therapies, thorax radiation therapy combined with other risk factors, such as preexisting cardiovascular disease, age, obesity, smoking, hypertension, diabetes and physical inactivity, may increase cardiovascular vulnerability [9].

At present, the most prevalent screening method is based on the periodic measurement of left ventricular ejection fraction (LVEF) before, during, and after chemotherapy with conventional 2-D transthoracic echocardiography [10]. Serial evaluation of LVEF is recommended for patients treated with anthracyclines or trastuzumab [11, 12]. Although several guidelines are available, there is not much scientific evidence about how often and how long cardiac function should be monitored during and after cancer treatment [13].

When LVEF decreases, there has already been considerable myocardial damage. Therefore, there is a need to investigate biomarkers that enable the early identification of cardiac deterioration. Early interval changes in individual biomarkers, such as ultrasensitive troponin I (TnI, cardiomyocyte injury), N-terminal proB-type natriuretic peptide (NT-proBNP, neurohormonal activation), and myeloperoxidase (MPO, oxidative stress) has been shown to be of incremental utility in identifying patients at risk for adverse outcomes with doxorubicin and trastuzumab [14].

As an increasing number of women survive breast cancer, the impact of cancer treatment in cardiac health is becoming ever more important. Since the early detection and treatment of cardiotoxicity can reduce its clinical effects, it is particularly important for oncologists to be aware of these side-effects and manage them appropriately. The purpose of this Delphi study is then to lead an agreed vision on the manner to improve on the definition, evaluation and management of chemotherapy-induced cardiac toxicity in breast cancer patients.

## Materials and methods

### Delphi study

The Delphi method is a process designed to achieve consensus in which expert opinion is gathered in a systematic way through multiple rounds of surveys [15]. Questioning takes place in rounds, and after each round, an anonymous summary of the responses is fed back to the group. Individual participants may then decide to keep their original answers or to change their opinion in the subsequent round of voting.

The Delphi panel (*n* = 100) was selected according to their clinical expertise on medical oncology, with a particular background in cardiotoxicity, and following a balanced geographical distribution all over Spain, of large academic hospitals and reference centers (52 % > 500-bed hospitals).

A modified two-round Delphi method was used in order to gather opinions and reach a consensus to prepare a list of recommendations to minimize cardiac toxicity in breast cancer patients. As an initial step, an evidence and best-practice scan of the literature was conducted by a small scientific board (5 investigators) with a mandate consistent with ameliorating the prevention, identification, and general management of cardiac toxicity in breast cancer therapy. The constructed recommendation statements were compiled to best reflect a range of pertinent issues and topics, and distributed to the entire panel for the first assessment of agreement. Panelists then received a personal invitation for an online questionnaire to rate each outcome. A 7-point Likert-type scale (1, totally disagree/never/never recommended; 4, uncertain or with objections to the question; 7, totally agree/always/always recommended) was used to measured agreement. The first survey (19 questions) enclosed five broad areas: profile of breast cancer patients; cancer therapy; and definition, evaluation and monitoring of cardiotoxicity. Updated statements were then carried out through a second online questionnaire, with first round scores displayed for each outcome. This round 2 questionnaire included then the frequency distribution of round 1 responses, each recipient’s own round 1 response and a list of previously submitted comments. Once more, participants were then asked to rate the statements, by using a 7-point Likert scale. The aim of this second round was to refine some of the most controversial round-1 questions, to gain more knowledge on those responses which brought about a bigger debate from the first questionnaire, and finally to revise and reach consensus on the pathway to manage cardiac toxicity in breast cancer patients.

### Data analysis and statistics

Data were analyzed globally and separately by the job position of each physician (head of service, head of section, assistant, others), and by hospital size (48 hospitals with ≤500 beds; 52 hospitals with >500 beds). Data are reported as mean values ± 1 SD (95 % CI).

In order to analyze the outcomes extracted from this two-round Delphi study, continuous variables were compared by Student’s *t* test (paired matched analysis), under assumption of normality distribution (Shapiro–Wilk test) or non-parametric alternative Wilcoxon signed-rank test, while categorical variables were studied through the McNemar–Bowker test of symmetry. This is a statistical test used on paired nominal data arranged into a *K* × *K* contingency table (*k* > 2) which compares the two versions of the survey.

Between-group comparisons (job positions and hospital size) were evaluated in order to evaluate possible differences by groups at the end of the study (2nd round). For continuous and normally distributed variables, a one-way analysis of variance, or the Kruskal–Wallis test, was carried out. This is a non-parametric method for testing whether samples originate from the same distribution that it is used for comparing two or more samples that are independent, and that may have different sample sizes. Categorical variables were compared by the Chi-square test or by the Fisher test. A two-tailed value of *p* of <0.05 was considered statistically significant.

## Results

### Participants

The Delphi panel consisted of 100 oncologists selected from different hospitals from the 17 different autonomous communities of Spain to ensure that regional differences were captured. A vast majority of panelists (76 %) were assistant physicians, most of them with >10-year experience (87 %) in breast cancer. Noteworthy, all participants asserted the availability to perform a CT scan at the hospital, followed by echocardiography (98 %) and biomarkers (86 %).

### Profile of patients

**Fig. 1:**
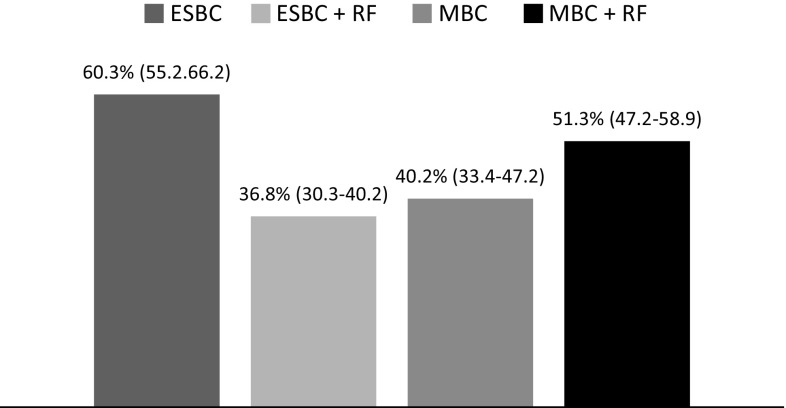
Current profile of breast cancer patients attended by the Spanish Delphi panel. Results are presented as a mean percentage (%; 95 % CI) of early-stage breast cancer patients (ESBC; 95 % CI 55–66); or metastatic breast cancer patients (MBC; 95 % CI 33.4–47.2) treated with chemotherapy, and those who refers at least one risk factor (RF) to develop cardiotoxicity (ESBC + RF, 95 % CI 30.3–40.2; MBC + RF, 95 % CI 47.2–58.9)

The Delphi panel was asked to rate the number of women with recently diagnosed breast cancer (*n* = 238). Sixty percent of the cases with early-stage breast cancer received chemotherapy, and 40 % for those with metastatic advanced disease (Fig. 1). Around 37 and 51.3 % of patients with early-stage and advanced metastatic breast cancer, respectively, had received chemotherapy and had at least one risk factor for cardiotoxicity (Fig. 1).

### Breast cancer therapy

**Fig. 2:**
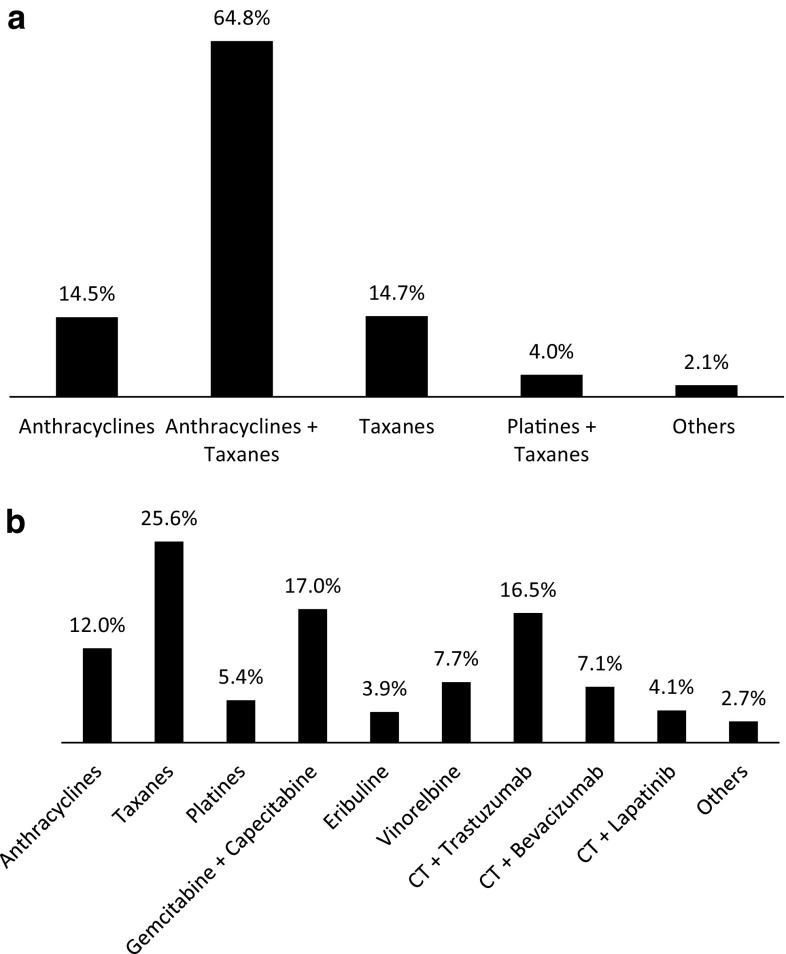
Frequencies of cancer therapy drugs used in early-stage (**a**) and metastatic breast cancer patients (**b**). *CT* chemotherapy

Chemotherapy can be an integral component of the adjuvant management strategy for women with early-stage breast cancer (Fig. 2a). Panelists showed that 80 % of patients received anthracyclines, either alone (64.8 %) or in combination with taxanes (14.5 %). In contrast, metastatic stage of breast cancer can be treated with a higher variety of anticancer drugs, and the treatment with anthracyclines represented a 12 % (Fig. 2b).

The patient with a diagnosis of cancer usually needs a rapid beginning of therapy, to avoid extension of the disease. In the current scenario, marked by an increase in long-term cancer survivors, it is essential to plan a strategy to managing cardiovascular disease associated with antineoplastic treatment. In this sense, a strong agreement was achieved between the participants to outline a plan from the very beginning of the therapy (data not shown), either being anthracyclines (mean of 6.3 based on a 7-point Likert scale) or chemotherapy plus trastuzumab (mean of 6.6).

### Cardiotoxicity

#### Definition of cardiotoxicity

Different definitions of cardiotoxicity have been historically used. One of the most common manifestations of cardiotoxicity associated with exposure to anticancer therapies is the development of left ventricular dysfunction (LVD) and overt congestive heart failure (CHF). The definition of LVD was proposed by the Cardiac Review and Evaluation Committee supervising trastuzumab clinical trials [16]. LVD is characterized by one or more of the following: (1) reduction of LVEF, either global or more specific and severe in the interventricular septum; (2) symptoms or signs associated with HF; (3) associated signs of CHF, including but not limited to S3 gallop, tachycardia, or both; and (4) decline in LVEF of at least 5 to <55 % in the presence of signs or symptoms of HF, or a reduction in LVEF ≥10 to <55 % without signs or symptoms of HF [19]. In the first round, participants commonly agreed to this classical definition of cancer therapeutics-related cardiac dysfunction (Likert score 5.9).

Recently, the American Society of Echocardiography along with the European Association of Cardiovascular Imaging outlined a consensus document that defines cardiac toxicity as a decrease in LVEF of >10 % points, to a value <53 % (normal reference value for 2D-echocardiography) [17]. This new definition also stated that LVD should be confirmed by repeated cardiac imaging at 2–3 weeks after the baseline diagnostic study showing the initial decline in LVEF. Furthermore, this consensus categorized LVD as *symptomatic* or *asymptomatic*, or with regard to reversibility: *reversible*, LVEF difference to <5 % of baseline; *partially reversible*, improved by ≥10 % points from the nadir but remaining >5 % below baseline; and *irreversible*, improved by <10 % points from the nadir and remaining >5 % below baseline [17]. The second round showed that our expert panel reached a very similar level of agreement for this new consensus definition (Likert score 5.8).

#### Clinical manifestations of cardiotoxicity induced by anthracyclines

Classically, three types of anthracycline-induced cardiotoxicity have been described: acute, chronic or delayed. Acute cardiotoxicity may occur during or immediately after a single dose of anthracycline treatment (present as transient arrhythmia and/or electrocardiographic changes, pericarditis–myocarditis syndrome and cardiac failure), which is usually reversible. Chronic progressive cardiotoxicity is clinically the most important type (present as dilated cardiomyopathy) and occurs the first year after the end of the treatment. It is dose-related, and may therefore be prevented with close monitoring and early treatment of subclinical cardiac dysfunction. Delayed cardiotoxicity may also be dose-related and occurs years to decades after exposure. Both chronic and delayed forms of cardiotoxicity were considered the most relevant by the panelists (Likert scores of 6.5 and 6.4, respectively).

#### Clinical relevance of risk factors for cardiotoxicity

Patients undergoing chemotherapy should have careful clinical evaluation and assessment of cardiovascular risk factors and comorbidities. All participants had the opportunity to score the importance as well as the need to identify treatment- and patient-related risk factors for cardiotoxicity in both early-stage and metastatic breast cancer.

Panelists asserted that the best predictor of cardiotoxicity was the total cumulative dose of anthracycline ≥450 mg/m^2^ (or its equivalent doses of epirubicin), either in early-stage (Table 1) or advanced disease (Table 2), followed by co-administration of additional agents and radiation therapy to the thorax. In both cases, throughout the second round, the panel identified the need of anthracycline-dosing schedule to be considered as a risk factor (*p* values round 1 vs. round 2: 0.0054 in early-stage disease and 0.0369 in metastatic disease).

**Table 1:** Data of the Likert scale mean values of the selected statements to rate the clinical importance (round 1) and the need for identification (round 2) of anthracycline- and patient-related risk factors for cardiac toxicity in early-stage breast cancer

Early-stage breast cancer	Importance (round 1)		Need (round 2)		Statistical differences (*p* value)		
	Participants (*n*)	Mean Likert score	Participants (*n*)	Mean Likert score	Round 1 vs. round 2	Job positions	Number of beds
Cardiac risk related to treatment							
Cumulative dose of anthracyclines ≥450 mg/m^2^	98	**6.7**	100	**6.7**	0.513	0.9513	0.8138
Combination of drugs	99	6.2	100	6.4	0.0845	0.4645	0.0067*
Thoracic radiation therapy	100	5.5	100	5.8	0.0188*	0.6155	0.6090
Anthracycline dose per cycle	100	4.9	100	5.5	0.0054*	0.1874	0.0377*
Cardiac risk related to patient							
Preexisting cardiac disease	98	**6.4**	100	**6.7**	0.0009*	0.1630	0.3307
Hypertension	100	5.4	100	5.8	0.0007*	0.050	0.6420
Age (>65 y.o.)	100	5.2	100	5.6	0.0016*	0.0051*	0.1844
Obesity	100	5.2	100	5.5	0.0164*	0.0060*	0.5195
Diabetes	100	5.1	100	5.6	0.0029*	0.0024*	0.9107
Smoking	100	4.8	100	5.3	0.0152*	0.0129*	0.7588
Hypercholesterolemia	100	4.4	100	5.0	0.0007*	0.0104*	0.6088
Physical inactivity	100	4.3	100	4.9	0.0029*	0.0024*	0.9107

Numbers in bold represent the highest degree of agreement according to the Likert used Scale (1–7)

Likert scale ranged from 1—not important/no need at all to 7—highly important absolutely needed. Comparisons between round 1 & 2 and among groups (job positions and hospital size) were also analyzed

* *p* < 0.05 was considered statistically significant

**Table 2:** Data of the Likert scale mean values of the selected statements to rate the clinical importance (round 1) and the need for identification (round 2) of anthracycline- and patient-related risk factors for cardiac toxicity in metastatic breast cancer

Metastatic breast cancer	Importance (round 1)		Need (round 2)		Statistical differences (*p* value)		
	Participants (*n*)	Mean Likert score	Participants (*n*)	Mean Likert score	Round 1 vs. round 2	Job positions	Number of beds
Cardiac risk related to treatment							
Cumulative dose of anthracyclines ≥450 mg/m^2^	100	**6.6**	100	**6.6**	0.1626	0.8235	0.1807
Combination of drugs	99	6.1	100	6.2	0.0116*	0.4579	0.0385*
Thoracic radiation therapy	100	5.6	100	5.6	0.0732	0.7561	0.1747
Anthracycline dose per cycle	100	5.1	100	5.5	0.0369*	0.4347	0.2501
Cardiac risk related to patient							
Preexisting cardiac disease	98	**6.3**	100	**6.2**	<0.0001*	0.1057	0.2025
Hypertension	100	5.4	100	5.4	0.0018*	0.0193*	0.5484
Age (>65 y.o.)	100	5.2	100	5.4	0.0022*	0.0026*	0.5832
Obesity	100	5.0	100	5.2	<0.0001*	0.0019*	0.8366
Diabetes	100	4.9	100	5.3	0.0004*	0.0241*	0.7770
Smoking	100	4.7	100	5.1	0.0007*	0.0308*	0.7393
Hypercholesterolemia	100	4.5	100	5.0	0.0003*	0.0019*	0.6892
Physical inactivity	100	4.4	100	4.8	0.0098*	0.0345*	0.7177

Numbers in bold represent the highest degree of agreement according to the Likert used Scale (1–7)

Likert scale ranged from 1—not important/no need at all to 7—highly important absolutely needed. Comparisons between round 1 & 2 and among groups (job positions and hospital size) were also analyzed

* *p* < 0.05 was considered statistically significant

When going through the patient-related risk factors, preexisting cardiovascular disease was clearly identified as a very important cardiac risk factor with similar results in early-stage cancer (Likert score 6.4) and metastatic cancer (Likert score 6.3). Other factors such as hypertension, age, diabetes, obesity, smoking, physical inactivity and hypercholesterolemia were also rated (Tables 1, 2).

#### Medical intervention to modify cardiotoxic risk-factors

**Fig. 3:**
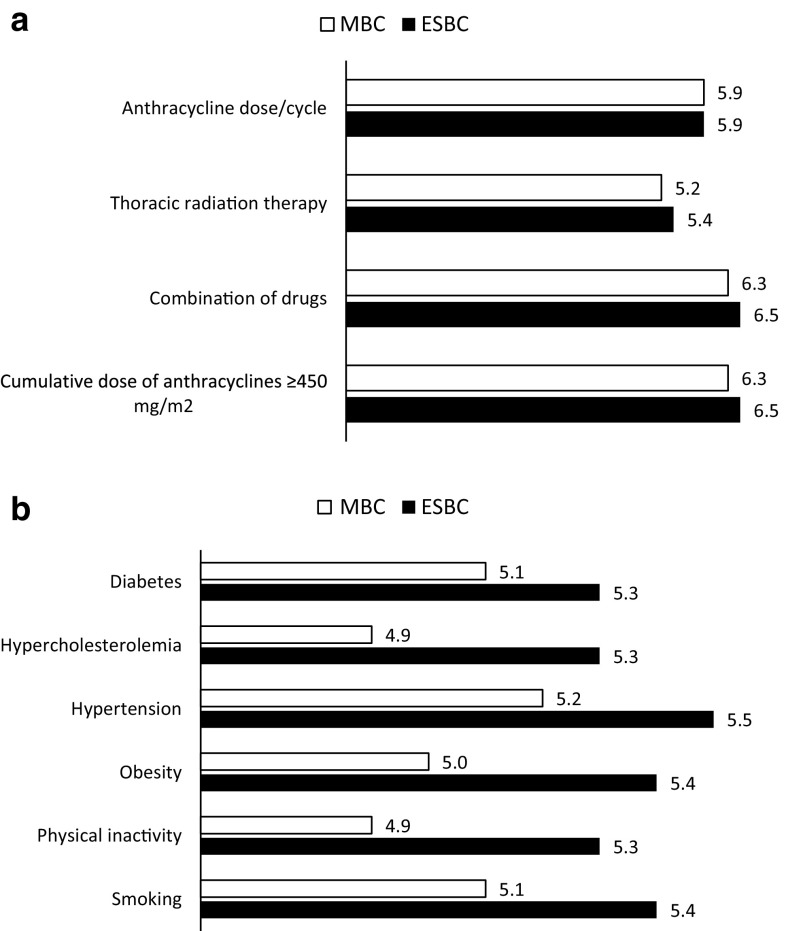
Graphic representation of the Likert scale mean values of the selected statements to rate the need for intervention of anthracycline-related risk factors (**a**) and patient-related risk factors (**b**) for cardiac toxicity in early-stage and metastatic breast cancer. Likert scale ranged from 1—no need at all to 7—absolutely needed. *ESBC* early-stage breast cancer, *MBC* metastatic breast cancer

Prior to treatment initiation, a risk–benefit analysis should be performed for each individual patient, including a thorough assessment in order to detect those conditions that may increase the risk of cardiac dysfunction during treatment. Of these, cumulative doses of anthracyclines and its potential combination with other anticancer drugs were deliberated as the most significant treatment-related risk factors susceptible to a medical intervention regardless the stage of the disease (Fig. 3a). In the second questionnaire, the dose of the anticancer drug administered during each session gained a significant acceptance among panel members as an important factor to be changed to avoid cardiotoxicity (Likert score of 4.9 in round 1 vs. 5.9 in round 2, for both early and advanced disease; *p* < 0.001). Classical cardiac patient-related risk factors (hypertension, smoking or hypercholesterolemia), fluctuated in a very short range of punctuation on the Likert scale with almost no difference regarding the stage of the disease (Fig. 3b).

#### Cooperation between oncologists and cardiologists

Oncologists must be fully aware of cardiovascular risks to prevent adverse cardiovascular effects of anticancer therapies. Likewise, cardiologists must now be ready to assist oncologists in the choice of therapy. Our study strongly evidenced that there is a real need for cooperation between these two areas. The vast majority of participants (96 %) agreed to develop a complementary labor, and approximately 91 % of them (data not shown) exposed the need to rely on a reference cardiologist who worked tightly with oncologists. They also stressed the need for a better availability for echocardiography (82.3 %) and a bit more than 40 participants supported the development of a novel unit, which could be termed cardio-oncology. The need of complementary education of the physicians was also outlined.

### Diagnosis of cardiotoxicity

**Fig. 4:**
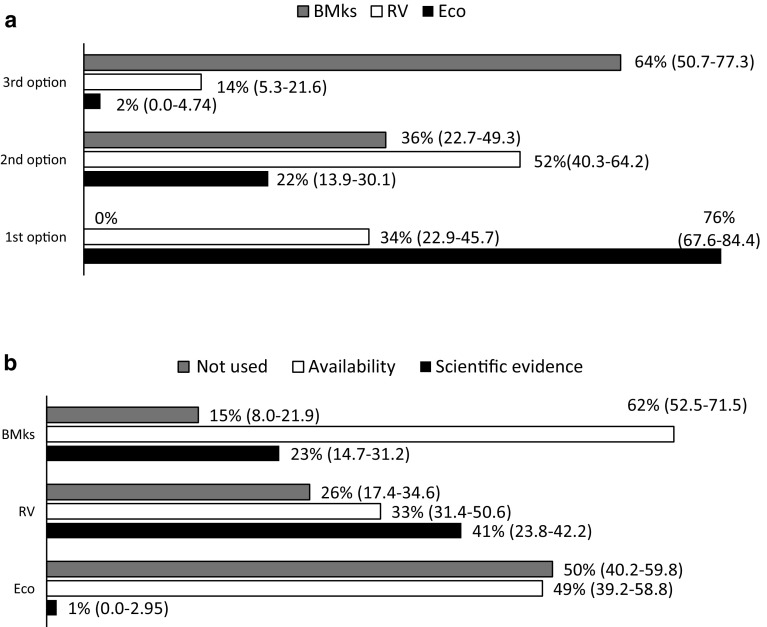
Preferential order of recommended techniques for serial monitoring of cardiac function during chemotherapy (**a**) and its practical use either by scientific evidence or availability, or not used (**b**). Results are presented as a mean percentage (95 % CI; %) of respondents (*n* = 100). *BMKs* biomarkers, *RV* radionuclide ventriculography, *Eco* echocardiography

Regular assessment of cardiac function is recommended by oncologic guidelines. Echocardiography (76 %) along with radionuclide ventriculography (52 %) were the proposed methods to monitoring changes in cardiac structure and function during chemotherapy (Fig. 4a). The use of other non-invasive methods, such as biological markers, was elected as the third option by 64 % of oncologists. This might be explained because testing biomarkers was the most readily available method to monitor cardiotoxicity among the hospitals represented by our Delphi panel (Fig. 4b).

### Evaluation and monitoring of cardiotoxicity

#### Early diagnosis of cardiotoxicity

Asymptomatic diastolic dysfunction may be an early finding in patients exposed to anthracycline therapy. Participants (99 %) agreed on a reduction in LVEF as the most prevalent early screening parameter of cardiac dysfunction (data not shown). In contrast, there was no consensus to bare the validity of using cardiac troponins in the follow-up of cardiotoxicity (Likert score 4.0).

#### Left ventricular ejection fraction monitoring

At present, the approach recommended by oncologic and cardiologic guidelines to detect breast cancer therapy-induced cardiac damage primarily relies on regular cardiac function monitoring (LVEF) at baseline and during the therapy. Our panel was requested to respond how often or how long cardiac function is currently monitored (what is done)/or should be monitored (what should be done) during and after cancer treatment (Table 3). Thirty-three and 78 % of our Delphi panel recommended the evaluation of LVEF at baseline and every 3 months until the end of the treatment with anthracyclines or chemotherapy plus trastuzumab, respectively, in those patients without previous risk factors. However, the presence of cardiotoxicity risks did not yield much difference the frequency of monitoring LVEF (anthracyclines, 27 % at baseline and chemotherapy plus trastuzumab, 75 % at baseline and every 3 months until the end of the therapy).

**Table 3:** Frequency of monitoring (currently done/should be done/recommended by expert consensus [20]) cardiac function during and after cancer therapy with anthracyclines or chemotherapy (CT) + trastuzumab, in breast cancer patients with (w) or without (w/o) cardiac risk factors

	What is done?				What should be done?				Expert consensus for multimodality imaging evaluation of adult patients during and after cancer therapy [20]	
	Anthracyclines (w/o) (%)	CT + trastuzumab (w/o) (%)	Anthracyclines (w) (%)	CT + trastuzumab (w) (%)	Anthracyclines (w/o) (%)	CT + trastuzumab (w/o) (%)	Anthracyclines (w) (%)	CT + trastuzumab (w) (%)	Anthracyclines	CT + trastuzumab
Symptoms-guide	21	2	2	–	9	–	1	1		
Baseline study	33	3	27	4	17	1	9	1		
Baseline + at 3 months	5	5	8	4	6	7	6	4		
Baseline every 3 months until completion of the therapy	19	78	28	75	23	75	33	69		100%
Baseline + end of therapy	17.0	3	22	1	21	6	14	2		
Baseline + end of therapy + follow-up	5	8	9	6	24	10	27	10	100 % (F/U at completion of treatment an 6-month later)	
More frequent controls	–	1	4	10	–	1	10	13	An alternative option: baseline echo, F/U with cardiac troponin I at each cycle, and ECHO 6 months after completion of treatment if TnI negative	

Results are expressed as a mean percentage (%) of respondents

#### Management of left ventricular ejection fraction

**Fig. 5:**
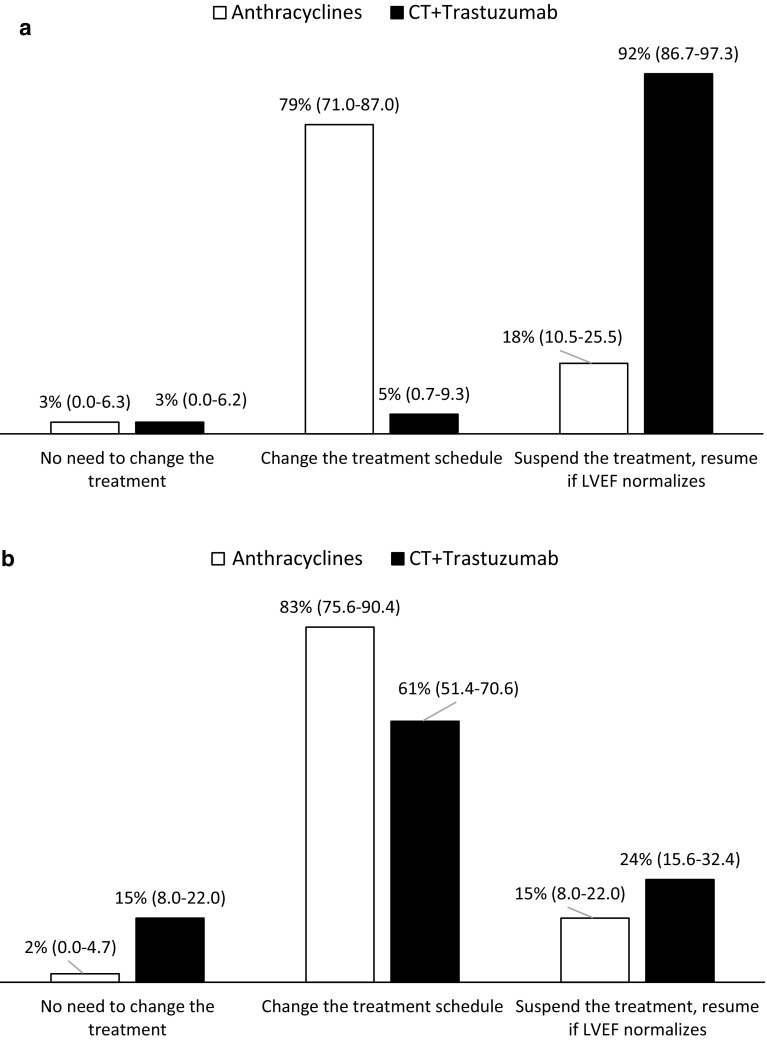
Strategies to minimize cardiotoxicity related to the cancer therapy (**a**) or to the treatment of cardiac dysfunction (**b**). Results are presented as a mean percentage (95 % CI; %) of respondents (*n* = 100). Cardiotoxicity was defined as a reduction of LVEF >10 % to below the normal limit LVEF <53 %

A decline of LVEF by more than 10 %, associated to an absolute LVEF value <53 % was suggested as a criterion for changing the dose schedule of anthracyclines, or suspending treatment of chemotherapy plus trastuzumab until the normalization of the left ventricular function (Fig. 5a). Additionally, it is mandatory to treat the cardiac dysfunction itself. Thus, in case of a reduction of LVEF >10 % to a value <53 %, most respondents stated that patient should be referred to the cardiologist either with anthracycline- or trastuzumab-induced cardiotoxicity (Fig. 5b).

#### Liposomal anthracyclines

**Fig. 6:**
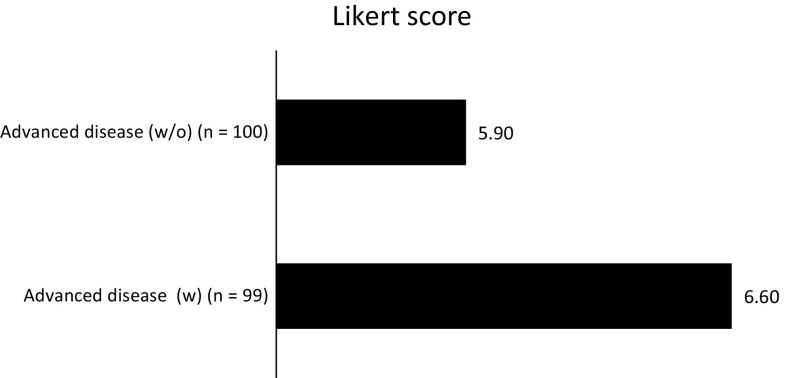
Evaluation for the need of liposomal anthracyclines in patients with advanced breast cancer, and with or without risk factors associated with cardiotoxicity (based on a 7-point Likert scale, where 1 means never recommended and 7 indicates always recommended)

Panelists agreed that cardiac safety of conventional anthracyclines is a long-term issue. This assumption was based on the fact of using an alternative therapy in case of worsening. Based on the present survey, liposomal anthracyclines should be considered as a treatment option for patients with metastatic breast cancer who are at cardiac risk (Fig. 6). The panelists believed that the use of liposomal anthracyclines is necessary in advanced breast cancer patients with (Likert score 6.6) or without risk factors associated with cardiotoxicity (Likert score 5.9).

## Discussion

Cardiotoxicity from breast cancer therapy is seen most commonly after treatment with anthracyclines and trastuzumab. Despite the large number of studies that have addressed the cardiotoxic effects from breast cancer therapy, there are a few recommendations for the detection, monitoring, and management of cardiotoxicity. This study assesses how to implement these recommendations in daily clinical praxis as well as the level of agreement of the experts with the guidelines.

The results of the Delphi panel show that Spanish oncologists generally follow the SEOM guidelines for treatment of early-stage or metastatic breast cancer patients (Fig. 2) [18, 19].

The individual choice of each adjuvant therapy must take into account the clinical benefit, patients preference and its possible side effects, mainly cardiotoxicity. Anthracyclines have been well studied in the curative setting for breast cancer with data showing the development of cardiomyopathy ranging from 1 to 7 % [20]. Since most studies and registries have not specifically analyzed anthracycline-induced cardiomyopathy among the several possible causes of chronic HF, formal estimates of the worldwide prevalence of anthracycline cardiotoxicity are lacking [21]. In this study, 38 and 51 % of women with early-stage and metastatic breast cancer, respectively, were found to have preexisting cardiac risk factors (Fig. 1).

The patient with a diagnosis of cancer usually needs a rapid beginning of therapy. The expert panel stated that when the therapy is established, it is necessary to plan rapidly the strategy to obtain the better goal of hitting the disease, reducing relevant toxicities as much as possible. Anthracyclines and the anti-HER2 trastuzumab used in chemotherapy are deeply studied, and the mechanism of action of their cardiotoxicity is well known. Anthracycline-induced injury has been described as “type I” cardiotoxicity, a dose-dependent, progressive, and generally thought to be irreversible toxicity [22]. Trastuzumab, though generally well tolerated, is associated with an infrequent but clinically significant risk of long-term cardiotoxicity. Unlike anthracyclines, trastuzumab induces a “type II” cardiotoxicity. The risk of damage is dose independent, generally reversible with discontinuation, and causes minimal ultrastructural changes [23]. The risk of developing trastuzumab-induced HF has been reported as 2–4 % when given alone, but as high as 27 % when administered with anthracyclines [24].

A clear definition of cardiotoxicity is still lacking [25]. The Delphi panel agrees with the Cardiac Review and Evaluation Committee to define cardiotoxicity as a decline in LVEF of at least 5 to <55 % in the presence of signs or symptoms of HF, or a reduction in LVEF above 10 to <55 % without signs or symptoms of HF. However, it should be borne in mind that it is difficult to assess a decline in LVEF of 5 % by two-dimension (2D) echocardiography since the rates of inter- and intra-observer variability are relatively high, and variations in LVEF ≥10 % are often found in the absence of any true modification [26].

Cardiac dysfunction associated with chemotherapy in breast cancer can be acute, subacute or chronic side effect. Findings from the Delphi panel suggest that there is still no clear difference between chronic and delayed forms of cardiotoxicity, since both forms are given the same degree of clinical relevance. Thus, there is a gap in knowledge about the prevalence of clinical and subclinical cardiac dysfunction in breast cancer long-term survivors who have been exposed to anthracyclines.

The present study also examines the risk factors for chemotherapeutics-associated cardiotoxicity and reviews current strategies followed by professionals to reduce the cardiotoxicity of anthracyclines and trastuzumab in the management of patients with early-stage and advanced breast cancer. A better understanding of these factors may help to reduce the occurrence and severity of cardiovascular side-effects. Thus, the best treatment-related predictor of cardiac toxicity seems to be the total cumulative dose of anthracycline ≥450 mg/m^2^ (or its equivalent doses of epirubicin), either in early-stage (Table 1) or advanced disease (Table 2). Extensive analyses have shown a direct relation between the occurrence of HF and the cumulative anthracycline dose. Early retrospective data indicate that the incidence of CHF is close to 3.0 % in patients receiving a cumulative doxorubicin dose of 400 mg/m^2^ increasing to 7.5 % at 550 mg/m^2^ and to 18.0 % at 700 mg/m^2^ [27]. Each dose of anthracycline appears to result in the death of cardiac myocytes. Despite the heart has well developed compensatory mechanisms, when these are overwhelmed, chronic dilated cardiomyopathy develops [28].

Other therapy-related risk factors outlined by the panel are the co-administration of additional agents and the radiation therapy to the thorax. It is well documented that anthracyclines and trastuzumab have synergistic effects on cardiac dysfunction when administered concomitantly [29, 30]. In a phase III trial [29], New York Heart Association (NYHA) III/IV HF was observed in 16 % of patients treated with trastuzumab concurrently with anthracycline and cyclophosphamide, compared with 3 % in those treated with cyclophosphamide and no limited dose of cumulative anthracyclines. In a similar trend, Romond et al. [30] reported that the incidence of cardiac death or CHF (NYHA III/IV) was greater in patients who received concurrent treatment with doxorubicin, cyclophosphamide, paclitaxel, and trastuzumab (4.0 %) than in patients who did not receive trastuzumab as part of their treatment regimen (1.3 %). After stopping trastuzumab, the majority of patients who experienced cardiac dysfunction recovered LVEF in the normal range. Furthermore, studies have shown that anthracycline-associated cardiac damage may become clinically more evident in patients who have already received cardiac injury from radiotherapy [31]. However, modern methods of breast/chest wall radiation, such as intensity-modulated radiation therapy, now avoid any appreciable dose to the heart in most patients.

Preexisting cardiovascular disease or cardiac risk factors is definitely identified as the most important predictor of cardiac toxicity after cancer therapy (Tables 1, 2). The presence of a cardiac risk factor may increase the chance of a patient experiencing a treatment related cardiac event, as suggested by an early retrospective study [32]. The probability of developing doxorubicin-induced CHF was higher in patients with previous cardiac disease or hypertension or both, although this was not statistically significant [20]. Likewise, at diagnosis, a substantial number of women with breast cancer are at significant risk of developing cardiovascular disease due to age and other major cardiac risk factors such as hypertension, hypercholesterolemia, diabetes, and obesity, which can adversely affect survival [5]. Surprisingly, it is noteworthy to observe the lower importance given to the easier factors to prevent with a better life-style (smoking, physical inactivity, hypertension, hypercholesterolemia).

Early identification of patients who are at risk for cardiotoxicity should be a primary goal for oncologists in the development of personalized cancer therapy. While cumulative doses of anthracyclines and its potential combination with other anticancer drugs remain to be as the most significant treatment-related risk factors susceptible to a medical intervention, dose limitation and schedule modification gain a significant acceptance among the Spanish oncologists as an important factor for the intervention (Fig. 3a). Indeed, the risk of anthracycline cardiotoxicity can be minimized by keeping the total lifetime cumulative dose of doxorubicin below the recommended threshold. It is generally recommended that the lifetime dose be <450 mg/m^2^ for doxorubicin and 900 mg/m^2^ for epirubicin. However, this approach results in the discontinuation of anthracycline.

With this Delphi study, it is still unclear whether or not classical cardiac patient-related risk factors (hypertension, smoking or hypercholesterolemia) may predispose more the oncologist for an intervention to reduce cardiovascular side effects. All the rates scored by the panel move in a very short range of consensus with almost no difference in the stage of the disease (Fig. 3b). In the review conducted by Seidman et al. [33], a multivariate analysis for potential risk factors, age, high arterial pressure and diabetes were significantly associated with the risk of developing cardiotoxicity, when doxorubicin was administered concomitantly with trastuzumab.

The improvement of diagnostic tools in both cardiology and oncology has led to an increased number of patients who have been treated for cancer and diagnosed with cardiovascular disease. As stated throughout this Delphi study, cardiologists and oncologists must therefore work together in an attempt to avoid or prevent adverse cardiovascular effects in patients from certain chemotherapies, especially for those who may be at a higher risk for such effects. Prevention of cardiotoxicity should begin before the initiation of cancer therapy, with the oncologist and the cardiologist working as a team: the oncologist performing a complete history and objective evaluation of the patient regarding cancer therapy or prevention, and the cardiologist evaluating cardiovascular parameters and function. The cardiovascular profile given by the cardiologist may help the oncologist in deciding the therapeutic approach, in terms of drug selection and schedule, for each individual patient. Therefore, it becomes apparent that the vast majority of oncologists from this panel agree to get academic training on cardiotoxicity (96 %), mainly through the organization of clinical sessions.

Clinically detectable cardiotoxicity is generally preceded by an interval of subclinical cardiac dysfunction. Therefore, techniques for early and reliable detection of LV dysfunction would help clinicians to identify patients at risk and predict future declines in cardiac function. In this study, oncologists emphasize the role of imagistic methods and biomarkers in assessment of chemotherapy-induced cardiotoxicity. As resulted from the survey, radionuclide ventriculography along with echocardiography, have been the proposed for monitoring changes in cardiac structure and function during chemotherapy (Fig. 4a). Radionuclide ventriculography (multiple uptake gated acquisition scan) is an established, well-validated, and widely used method to determine ejection fraction [34]. Results from a recent retrospective analysis suggest that an incipient fall in LVEF detected on serial equilibrium radionuclide ventriculography during doxorubicin therapy provides an appropriate and cost-effective approach for predicting and preventing impending CHF [35]. Echocardiography is a widely used non-invasive method of monitoring cardiotoxicity of cancer therapy that provides a wider spectrum of information on cardiac morphology and function [34]. With modern echocardiography, it is possible to obtain images to detect early subclinical myocardial injury.

Based on current data, it has been proposed that biomarker monitoring of patients during anthracycline exposure may provide crucial evidence of early cardiac damage [14]. Strong data indicate that troponin offers the ability to detect chemotherapy-induced cardiotoxicity in its earliest phase, before the reduction in LVEF [36]. However, these biomarkers are still not routinely measured. Actually, the determination of cardiac troponins has been the third choice of panelists; this result could be explained because testing biomarkers was the most readily available method for the assessment of cardiotoxicity among the hospitals represented by our Delphi panel (Fig. 4b).

Cardiologic surveillance is required during chemotherapy, to select the oncologic regimen that would achieve the highest possible rate of cure or remission, with the lowest possible rate of treatment withdrawals and cardiovascular side effects. Findings from this study reveal that around half of the panel agree upon controlling LVEF at baseline, and 19 % additionally every 3 months and thereafter until the end of anthracycline therapy, and this schedule is not related whether the presence or absence of cardiovascular risk factors (Table 3). Ideally, results are not much different. There is a strong variability among respondents at choosing the monitoring schedule of cardiac function in breast cancer treated with anthracyclines. However, to date, there are no evidence-based guidelines for cardiotoxicity monitoring during and after anticancer therapies in adults [13]. The goal of safety monitoring during trastuzumab administration is to detect cardiac dysfunction early, before the development of clinical symptoms, when therapy can be provided to prevent permanent changes. The trastuzumab package insert calls for monitoring of LVEF at baseline/pretreatment and at 3- to 4-month intervals throughout trastuzumab treatment, in combination with clinical evaluation for symptoms of HF as standard practice [37]. This is also the option of our Delphi panel (Table 3).

Primary goals of prevention are to minimize the cardiac toxicity of chemotherapy while maintaining its oncological efficacy. There are different strategies addressed to decrease cardiotoxicity in patients receiving chemotherapy: changing the dose of the drug, alternative scheduling techniques, or completely eradicating the use of the therapy. A decline of LVEF by more than 10 %, associated to an absolute LVEF value <50–55 % has been suggested as a criterion for suspending the treatment [38]. Facing such condition, our results underscore the need of changing the treatment schedule of anthracyclines. Many oncologists (92 %) chose the evidence-based option to “discontinue trastuzumab, resume if LVEF normalizes” (Fig. 5a). Indeed, in some clinical studies reporting reduced LVEF <40 % following trastuzumab therapy, discontinuation of trastuzumab (without additional intervention) was sufficient to prevent cardiac events [39]. Additionally, this Delphi study demonstrates that, in the presented case of an LVEF decline >10 % to below the institutionally defined normal value (53 %), patient should be transferred to a cardiologist regardless the cancer therapy regimen (Fig. 5b). More concerning is that 15 % of the oncologists would suggest not to do anything in those patients treated with chemotherapy plus trastuzumab, thus depriving them of potentially lifesaving cardiac therapy.

Finally, the expert panel acknowledged that cardiac safety of conventional anthracyclines is a long-term issue, so that the use of liposomal anthracyclines should be considered as a therapeutic option for patients with metastatic breast cancer who are at cardiac risk (Fig. 6). The encapsulation of a cytostatic agent within a macromolecular vector, such as a liposome, significantly reduces its distribution volume, diminishing its diffusion and consequently, the toxicity for healthy tissues while increasing the concentration within the neoplastic tissue [40]. The clinical opinion of the majority of respondents in this two-round survey are supported by the available literature. Clinical trials have shown the two possible formulations of liposomal doxorubicin (nonpegylated and pegylated) to have similar efficacy with less cardiac toxicity when compared with free doxorubicin [41–43]. In a phase III trial of 509 women with metastatic breast cancer, pegylated liposomal doxorubicin 50 mg/m^2^ (every 4 weeks) provided comparable efficacy to free doxorubicin 60 mg/m^2^ (every 3 weeks), with significantly reduced cardiotoxicity, even in those subgroups at increased cardiac risk (≥65 years of age, prior adjuvant anthracycline, cardiac risk factors) [41]. Two randomized phase III trials have shown that nonpegylated liposomal doxorubicin was significantly less cardiotoxic than conventional doxorubicin in the first-line treatment of metastatic breast cancer (13 vs. 29 % patients, *p* = 0.0001 [42]; 6 vs. 21 % patients, *p* = 0.0001 [43]), while providing comparable antitumour activity (response rate 26 % in both groups [42]; 43 % in both groups [43]).

Based on the above data, liposomal doxorubicin should be considered as a treatment option for patients with metastatic breast cancer who are at cardiac risk. Furthermore, nonpegylated liposomal doxorubicin may have efficacy as well as cardiotoxicity advantages over free doxorubicin for patients treated adjuvantly with anthracyclines.

This study has several limitations. Firstly, data were generated through a Delphi process and not collected in prospective or retrospective studies. Secondly, we were unable to compare results with other studies, as this study was the first of its kind in cardiac toxicity in oncology. Thirdly, the survey design forced respondents to select answers in a multiple-choice format, and respondents were limited to the choices provided. And last but not least, the key limitation with any consensus approach is the experts included and their opinion and biases [18]. There is no ideal size for participants in a Delphi survey. Smaller groups tend to be more homogeneous, resulting in a potentially limited view of consensus, whereas larger groups often have a range in depth of expertise resulting in the development of only general statements that achieve consensus [18].

## Conclusions

This Spanish survey was conducted to gain a better understanding of the knowledge base and clinical opinions of oncologists involved in the treatment of cancer patients being treated with potentially cardiotoxic therapy. To our knowledge, this is the first study of this kind in the field of cardiac oncology and highlights many controversial clinical issues within the field.

Around 80 % of breast cancer patients in Spain are treated with anthracyclines, whose cardiotoxic effect is very well known, and a high percentage of those have strong cardiovascular risk. Cardiac function should also be accurately assessed before starting the therapy; for high-risk patients with a good prognosis, repeat assessments during and after treatment should also be considered. Furthermore, the elected method of assessment should be one with which the center is familiar. Our panel concluded that echocardiography and radionuclide ventriculography (less risk of radiation) are the most frequent imaging techniques used across Spain. LVEF measured at baseline and every 3 months thereafter until completion of treatment in women with breast cancer treated either with anthracyclines or chemotherapy plus trastuzumab may help oncologists to identify an early diagnosis of ventricular dysfunction, even if asymptomatic, and to guide the clinician on the subsequent treatment plan in terms of therapy adjustment, closer follow-up of cardiac function, and appropriateness of cardiovascular therapy.

This report also evidences the need to develop a complementary labor between cardiologists and oncologists, and stresses the need for a better availability for echocardiography and the use of new biomarkers in the clinical practice. In fact, the development of a novel unit, which could be termed cardio-oncology, has been proposed. And this unit should be integrated by engaged professionals who manage optimally material resources for a better care of patients.

Strategies to prevent and manage chemotherapy-induced cardiotoxicity are important for all breast cancer patients. Ideally, these should be initiated before therapy, in order to minimize the possibility of irreversible cardiac damage. In addition to screening patients for patient and treatment-related cardiovascular risk factors, proactive treatment of modifiable risk factors should also be undertaken. Primary goals of prevention are to minimize the cardiac toxicity of chemotherapy while maintaining its oncological efficacy. This study underscored the need of changing the treatment schedule of anthracyclines, or suspending the treatment of chemotherapy plus trastuzumab until the normalization of the left ventricular function. Finally, Spanish oncologists have also highlighted the advantages of using liposomal anthracyclines instead.

The challenge for the future will be to develop methods for early detection of cardiac dysfunction, identify strategies for prevention and treatment of cardiotoxicity, and establish clinical guidelines for practicing physicians. Many questions remain unanswered, and ongoing research and collaboration between oncologists and cardiologists are needed to ensure optimal efficacy and safety of current and future anticancer agents.
